# Nurse-led HIV services and quality of care at health facilities in Kenya, 2014–2016

**DOI:** 10.2471/BLT.16.180646

**Published:** 2017-04-05

**Authors:** Miriam Rabkin, Matthew Lamb, Zainab T Osakwe, Peter R Mwangi, Wafaa M El-Sadr, Susan Michaels-Strasser

**Affiliations:** a International Center for AIDS Care and Treatment Programs (ICAP) at Columbia University, 722 West 168^th^ Street, New York, NY 10032, United States of America.; b ICAP at Columbia University, Nairobi, Kenya.

## Abstract

**Objective:**

To develop a novel measure to characterize human immunodeficiency virus (HIV) programme quality at health facilities in Kenya and explore its associations with patient- and facility-level characteristics.

**Methods:**

We developed a composite indicator to measure quality of HIV care, comprising: assessment of eligibility for antiretroviral therapy (ART); initiation of ART; and retention on ART or in care, if ineligible for ART, for 12 months. We applied the comprehensive retention indicator to routinely collected clinical data from 13 331 patients enrolled in HIV care and treatment at 63 health facilities in the Eastern and Nyanza regions of Kenya from 1 January 2014 to 31 March 2016. We explored the association between facility- and patient-level characteristics and the primary outcome: appropriate staging and management of HIV, and retention in care over 12 months.

**Findings:**

Of the enrolled patients, 8404 (63%) achieved comprehensive retention 12 months after enrolment in care. In univariate analyses, patients at facilities where nurses delivered HIV treatment services (including eligibility assessment, initiation and follow up of ART) had significantly higher comprehensive retention rates at 12 months. In multivariate analyses, after adjusting for both facility- and patient-level characteristics, patients at facilities where nurses initiated ART had significantly higher comprehensive retention in care at 12 months (relative risk, RR: 1.22; 95% confidence interval, CI: 1.00–1.48).

**Conclusion:**

Nurse-led HIV services were significantly associated with quality of care, confirming the central role of nurses in the achievement of global health goals, and the need for further investment in nursing education, training and mentoring.

## Résumé

**Objectif:**

Concevoir une nouvelle mesure pour caractériser la qualité du programme VIH (virus de l'immunodéficience humaine) dans des centres de santé au Kenya et analyser ses corrélations avec les caractéristiques des patients et les caractéristiques des centres de santé.

**Méthodes:**

Pour mesurer la qualité des soins anti-VIH, nous avons conçu un indicateur composite, qui englobe: l'évaluation de l'éligibilité à une thérapie antirétrovirale (TAR), le lancement de la TAR et le continuum de la TAR ou -pour les patients non éligibles à une TAR- le continuum de soins. Nous avons appliqué cet indicateur de continuum complet aux données cliniques habituellement consignées par les centres de santé; des données qui ont porté sur 13 331 patients pris en charge et traités pour une infection à VIH dans 63 centres de santé de la province orientale et de la province de Nyanza au Kenya entre le 1^er^ janvier 2014 et le 31 mars 2016. Nous avons analysé les corrélations entre les caractéristiques des centres et des patients et le principal critère observé: le correct enchaînement des étapes / la prise en charge appropriée de l'infection à VIH et le continuum de soins sur 12 mois.

**Résultats:**

Le critère de continuum complet 12 mois après la prise en charge a été satisfait pour 8 404  patients (63%). Dans nos analyses univariées, les taux de continuum complet à 12 mois ont été sensiblement supérieurs chez les patients pris en charge dans des centres où les services de traitement contre le VIH ont été menés par des infirmiers (chargés notamment de l'évaluation de l'éligibilité à une TAR ainsi que du lancement et du suivi des TAR). Dans nos analyses multivariées, après ajustement en fonction des caractéristiques des centres et des patients, des taux sensiblement supérieurs de continuum complet à 12 mois ont été observés chez les patients pris en charge dans des centres où les TAR ont été lancées par des infirmiers (risque relatif: 1,22; intervalle de confiance de 95%: 1,00–1,48).

**Conclusion:**

Les services anti-VIH menés par des infirmiers ont largement été associés à une bonne qualité des soins, ce qui confirme le rôle central des infirmiers dans l'atteinte des objectifs de santé mondiaux et la nécessité d'investir davantage dans l'apprentissage, la formation et le tutorat des infirmiers.****

## Resumen

**Objetivo:**

Desarrollar una medida novedosa para caracterizar la calidad del programa contra el virus de la inmunodeficiencia humana (VIH) en centros sanitarios de Kenya y explorar sus asociaciones con las características a nivel de paciente y centro.

**Métodos:**

Se desarrolló un indicador compuesto para medir la calidad de la atención contra el VIH formado por: una evaluación de la elegibilidad para el tratamiento antirretrovírico (TAR), la iniciación del TAR y la retención en el TAR o en la atención, si no es posible recibir el TAR, durante 12 meses. Se aplicó el indicador de retención completo a datos clínicos recopilados de forma rutinaria de 13 331 pacientes inscritos en la atención y el tratamiento contra el VIH en 63 centros sanitarios en las provincias del Este y Nyanza, en Kenia, del 1 de enero de 2014 al 31 de marzo de 2016. Se analizó la asociación entre las características a nivel de pacientes y de centros y el resultado principal: una estadificación y gestión adecuadas del VIH, así como una retención de la atención durante 12 meses.

**Resultados:**

De los pacientes inscritos, 8 404 (63%) lograron una retención completa al cabo de 12 meses de inscribirse en la atención. En análisis univariables, los pacientes de los centros donde las enfermeras ofrecieron los servicios del tratamiento contra el VIH (incluida una evaluación de la elegibilidad, la iniciación y el seguimiento del TAR) registraron unas tasas de retención completa mucho más altas en 12 meses. En análisis multivariables, tras ajustar las características a nivel de pacientes y de centros, los pacientes de los centros donde las enfermeras iniciaron el TAR registraron unas tasas de retención completa mucho más altas en 12 meses (riesgo relativo (RR): 1,22; intervalo de confianza (IC) del 95%: 1,00–1,48).

**Conclusión:**

Los servicios contra el VIH dirigidos por enfermeras estaban significativamente asociados con la calidad de la atención, lo que confirma el importante papel de las enfermeras a la hora de lograr objetivos de salud globales, así como la necesidad de invertir más en la educación, formación y orientación de las enfermeras.

## ملخص

**الغرض:**

وضع مقياس جديد لتوصيف جودة البرنامج الخاص بالتعامل مع فيروس عوز المناعة البشري (HIV) في المرافق الصحية بكينيا واكتشاف ارتباطاته بالخصائص القائمة على مستوى المريض ومستوى المِرفَق الصحي.

**الطريقة:**

قمنا بوضع مؤشر مركّب لقياس جودة رعاية فيروس عوز المناعة البشري، والذي يشمل: الوقوف على درجة الأهلية لتلقي العلاج بمضادات الفيروسات القهقرية (ART)؛ وبدء العلاج به؛ والاستمرار في تقديم العلاج به أو متابعة تقديم الرعاية لمدة 12 شهرًا في حال عدم الأهلية لتلقي العلاج بمضادات الفيروسات القهقرية. قمنا بتطبيق مؤشر الاستبقاء الكامل لجمع البيانات السريرية بشكل روتيني من 13,331 مريضًا مسجلاً في برنامج رعاية المصابين بفيروس عوز المناعة البشري وعلاجه في 63 مرفقًا صحيًا في المناطق الشرقية ومنطقة نيازا بكينيا في الفترة من 1 يناير/كانون الثاني 2014 إلى 31 مارس/آذار 2016. واكتشفنا الارتباط ما بين الخصائص القائمة على مستوى المرضى ومستوى المرافق الصحية، وتمثلت النتيجة الأولية فيما يلي: التدريج الملائم وإدارة فيروس عوز المناعة البشري، والاستمرار في تلقي الرعاية على مدار 12 شهرًا.

**النتائج:**

من بين المرضى المسجلين، استمر 8404 مرضى (بنسبة 63‏‏%) في تلقي ميزة الاستبقاء الشامل في برنامج العلاج لمدة 12 شهرًا بعد التسجيل في الرعاية. في التحاليل أحادية المتغير، تمتع المرضى المترددون على المرافق الصحية التي توفر فيها أطقم التمريض خدمات علاج فيروس عوز المناعة البشري (بما في ذلك تقييم مدى استحقاق المريض لتلقي مضادات الفيروسات القهقرية وبدئها ومتابعتها) بمعدلات أعلى بشكلٍ ملحوظ لاستبقائهم في برامج العلاج بمرور 12 شهرًا. أما في التحاليل متعددة المتغيرات، فبعد تعديل الخصائص على مستوى المرضى ومستوى المرافق الصحية على حدٍ سواء، فقد تبين أن المرضى المترددون على المرافق التي تقدم فيها أطقم التمريض العلاج مضادات الفيروسات القهقرية بمعدلات أعلى بشكلٍ ملحوظ للاستمرار في تلقي الرعاية الشاملة بمرور 12 شهرًا (بلغت نسبة الاختطار النسبي: 1.22؛ وبنسبة أرجحية مقدارها 95‏%: 1.00–1.48).

**الاستنتاج:**

ارتبطت خدمات فيروس عوز المناعة البشري المقدمة من أطقم التمريض بجودة الرعاية بشكلٍ ملحوظ، مما يؤكد على الدور الفعال لأطقم التمريض في تحقيق الأهداف الصحية العالمية، والحاجة إلى المزيد من الاستثمار في تعليم الممرضين وتدريبهم وتوجيههم.

## 摘要

**目的：:**

旨在制定一种新型衡量指标，以表征肯尼亚医疗机构中人类免疫缺陷病毒（艾滋病毒）计划的质量并探索其与患者和医疗机构层面特征的相关性。

**方法：:**

我们制定了一个综合指标来衡量艾滋病毒护理质量，包括： 评估符合抗逆转录病毒治疗 (ART) 的条件；开始进行 ART；以及 12 个月中 ART 或护理（如果不符合 ART 条件）中的留存率。 我们采用了全面留存指标定期收集了 2014 年 1 月 1 日至 2016 年 3 月 31 日期间在肯尼亚东部和尼安萨地区 63 个医疗机构中接受艾滋病毒护理和治疗的 13 331 名入选患者的临床数据。我们探索了医疗机构和患者层面的特征以及主要结果： 适当的艾滋病毒分期和管理，以及 12 个月中护理留存率。

**结果：:**

入选患者中，8404 人 (63%) 在接受治疗后的 12 个月中实现全面留存。 在单变量分析中，由护士提供艾滋病毒治疗服务（包括条件评估、开始进行 ART 和随诊）的医疗机构中的患者在 12 个月中的全面留存率显著较高。 在多变量分析中，调整医疗机构和患者层面特征之后，护士开始进行 ART 治疗的医疗机构中的患者在 12 个月中的全面留存率显著较高（相对危险度： 1.22；95% 置信区间： 1.00–1.48)。

**结论：:**

由护士主导的艾滋病毒防治服务与护理质量显著相关，证实了护士在实现全球卫生目标方面所起的核心作用，以及进一步投资护理教育、培训和指导的必要性。

## Резюме

**Цель:**

Разработать новые критерии определения качества программы помощи при иммунодефиците человека (ВИЧ) в медицинских учреждениях Кении и изучить ее взаимосвязь с характеристиками на уровне пациента и уровне медицинского учреждения.

**Методы:**

Мы разработали комбинированный показатель для измерения качества медицинской помощи при ВИЧ, включающий оценку приемлемости антиретровирусной терапии (АРТ), начало АРТ, а также удержание на АРТ или в системе оказания медицинской помощи, если пациент не подлежит АРТ, в течение 12 месяцев. Мы применили комплексный показатель удержания к собранным по стандартной методике клиническим данным от 13 331 пациента, включенного в систему ухода и лечения ВИЧ-инфекции, в 63 медицинских учреждениях в Восточной провинции и в провинции Ньянза в Кении с 1 января 2014 года по 31 марта 2016 года. Мы изучили взаимосвязь между характеристиками на уровне медицинского учреждения и на уровне пациента и первичным результатом: правильное определение стадии и надлежащее лечение ВИЧ, а также удержание в системе оказания медицинской помощи в течение 12 месяцев.

**Результаты:**

Из зарегистрированных пациентов 8404 человека (63%) достигли полного удержания в течение 12 месяцев после включения в систему оказания медицинской помощи. При однофакторном анализе пациенты в медицинских учреждениях, где медсестры оказывали медицинские услуги по лечению ВИЧ (включая оценку приемлемости, начало и последующее применение АРТ), имели значительно более высокие уровни полного удержания в течение 12 месяцев. При  многофакторных анализах после корректировки характеристик как на уровне медицинского учреждения, так и на уровне пациента пациенты в тех медицинских учреждениях, где медсестры начали АРТ, имели значительно более высокое полное удержание в системе оказания медицинской помощи в течение 12 месяцев (относительный риск: 1,22; 95%- й  доверительный интервал, ДИ: 1,00–1,48).

**Вывод:**

Сестринское обслуживание при ВИЧ-инфекции в значительной степени связано с качеством медицинской помощи, что подтверждает центральную роль медсестер в достижении глобальных целей в области здравоохранения и необходимость дальнейших инвестиций в сестринское образование, обучение и наставничество.****

## Introduction

The global response to the human immunodeficiency virus (HIV) epidemic has been remarkably successful. In low- and middle-income countries, the number of people living with HIV who have access to antiretroviral treatment (ART) rose from 400 000 in 2003 to 17 million in 2015. Modelling estimates from the Joint United Nations Programme on HIV/AIDS (UNAIDS) suggest that annual deaths from the acquired immunodeficiency syndrome (AIDS) have dropped by 43% over the same period^1^ and 7.8 million deaths have been averted by the scale-up of ART services.^2^ Increased access to prevention and treatment has also led to an estimated 35% drop in new HIV infections since 2000, including a 58% decrease among children.^3^

Despite these achievements, UNAIDS modelling highlights that only 46% of people living with HIV have initiated ART and 2.1 million new HIV infections occurred in 2015.^1^ Scaling up HIV prevention and treatment services and ensuring their quality is important if the ambitious global goals are to be met.^4^ The 2020 UNAIDS 90–90–90 targets call for 90% of people living with HIV to be aware of their HIV status, 90% of those diagnosed with HIV to be on ART and 90% of those on ART to achieve viral suppression.^5^ To reach these targets, people living with HIV need to learn their HIV status, link to appropriate treatment, achieve virological suppression and remain in care and on treatment for their lifetime. Careful attention to each step along the treatment continuum is essential,^6^ as sustained retention in care remains a challenge in many HIV programmes.^7^

HIV programme quality is assessed via diverse process indicators in both low- and high-resource settings.^8^ For example, the Site Improvement through Monitoring System approach used by the United States President’s Emergency Plan for AIDS Relief (PEPFAR) assesses core essential elements for quality service delivery at the community, clinic (site) and above-site levels. HIV programme quality is often also assessed by examining outcomes along the continuum of HIV care, including: the proportion of patients testing positive for HIV who are linked to care; the proportion started on treatment; the proportion who remain in care; and the proportion who achieve viral suppression (in settings where viral load measurement is available). Because this approach only includes patients eligible for ART, more inclusive measures of the continuum of HIV care have been proposed that capture outcomes for all HIV-infected individuals enrolled in care.^9^

We used a novel and comprehensive measure of comprehensive retention in HIV care to characterize programme quality at health facilities in Kenya, and explored the associations with patient- and facility-level characteristics.

## Methods

### Study design and setting

We conducted an observational, longitudinal analysis using routinely collected clinical information on 13 331 HIV-infected individuals enrolled at 63 health facilities in the Eastern and Nyanza regions of Kenya. Health facility staff provided clinical services according to Kenya’s national guidelines.^10^ All facilities were receiving technical assistance from the International Center for AIDS Care and Treatment Programs (ICAP) at Columbia University via funding from PEPFAR; this included in-service training using Kenya’s national training curricula, supportive supervision, and mentoring on routine data collection and analysis. For this analysis, we assessed the relationship between selected patient and facility parameters and the outcome indicator: comprehensive retention in HIV care.

### Data sources

#### Patient-level data

The Optimal Models of HIV care project is a multi-country project, funded by the Centers for Disease Control and Prevention, aimed at improving the use of routinely-collected HIV care and treatment data for programme monitoring, evaluation, operations research and implementation science. As part of this project, we collect de-identified patient-level data from ICAP-supported health facilities in Ethiopia, Kenya, Mozambique, Rwanda and the United Republic of Tanzania. Details of the methods are described elsewhere.^11^ Briefly, at each patient visit, clinic staff document routinely collected patient information on national forms. This information is regularly entered into electronic databases by data clerks, with data quality assessments performed at least annually at each clinic. This analysis focused on Kenya, chosen because of the variability in nurse-led ART services in the country and to restrict between-country variability as an explanation for any findings. Data were collected on patients enrolling in HIV care between 1 January 2014 and 31 March 2015. Information on follow-up visits was included up to 31 March 2016.

Patient-level covariates included age (categorized into < 2 years, 2–14 years, 15–24 years, 25–39 years, 40–49 years and ≥ 50 years), sex and HIV disease stage at enrolment. Disease stage was categorized into: CD4+ T lymphocyte (CD4+ cell) count < 200 cells/µL, or WHO stage 3 or 4;^12^ CD4+ cell count > 200 cells/µL, or WHO stage 1 or 2; or missing data on both CD4+ cell and WHO staging.

#### Facility-level data

ICAP staff collected facility-level characteristics in August 2015 using ICAP’s Program and Facilities Characteristics Tracking System site checklist. This is a biannual site assessment of the HIV care and treatment services and staffing at health facilities supported by ICAP.

Facility-level factors included: facility type (public primary, public secondary or private); setting (urban, semi-urban or rural); availability of on-site CD4+ cell testing; patient volume (categorized based on number of new enrollees into HIV care per year); availability of specific services (community ART adherence groups; financial incentives for adherence; facility-based group counselling for adherence support); and types of health-care worker at the facility (physicians, nurses, clinical officers [non-physician clinicians] or outreach workers). In addition, the survey assessed the availability of nurse-led ART services (assessment of eligibility for ART; initial prescriptions for ART; and follow-up care for ART).

### Outcome definition

The primary outcome of interest in this analysis was a comprehensive measure combining retention in HIV care for 12 months and receipt of key services during that time. The comprehensive retention indicator included variables relevant to patients who were eligible for ART as well as those who were not yet eligible for ART; all patients received clinical and immunological monitoring, prophylaxis for opportunistic infections and counselling.

Patients who started ART within 12 months of enrolment met the definition of comprehensive retention in HIV care if they were assessed for ART eligibility; initiated on ART or retained in care until becoming eligible for ART (based on country guidelines),^10^ and retained on ART for 12 months after enrolment at the health facility. Patients also met the definition of comprehensive retention in care if they were assessed for ART eligibility; found to be ineligible based on country guidelines; and retained in care at the health facility for 12 months after enrolment. Evidence of assessment for eligibility was a record of CD4+ cell count or World Health Organization (WHO) HIV/AIDS clinical stage^12^ before ART initiation.

Patients did not meet the definition of comprehensive retention if they were missing a recorded WHO stage or CD4+ cell count in their medical records; designated as ART-eligible (according to CD4+ cell count or WHO stage); and did not initiate ART. Patients not in comprehensive retention also included those who died or became lost to follow-up in the 12 months after enrolment.

For all analyses, patients were considered lost to follow-up if they had no recorded visit in the last 3 months (patients on ART) or 6 months (patients not on ART).

### Statistical methods

Descriptive statistics were used to describe the demographic and clinical characteristics of the study population. In analyses investigating factors associated with achievement of retention in care at 12 months (as defined above), univariate analyses were conducted using generalized linear mixed log-binomial regression models with random intercepts to account for within-clinic correlation. Outcomes are presented as the relative risk (RR) and 95% confidence interval (CI) of achieving 12-month retention in HIV care.

For the multivariable analyses, two models were examined. The first model included only facility-level covariates, while the second model included facility-level and patient-level covariates. For models 1 and 2, facility type was included because it was a priori identified as a potential confounder of the relationship between the availability of nurse-led initiation/management of ART and retention at 12 months. Other facility-level covariates found significant at α < 0.05 in the univariate analysis were also included in the multivariable models. The three measures focusing on nurse-led initiation and management of ART (i.e. assessment of ART eligibility by nurse; initiation of ART by nurse; and follow-up of ART by nurse) were highly collinear with each other; therefore only one (initiation of ART by nurse) was included in the multivariable models. The latter was selected because it had the strongest association with our outcome of interest in the univariate analysis. In model 2, patient-level covariates included age, sex and disease stage at enrolment in care.

### Ethical approval

Use of anonymized patient-level data from health facilities was conducted as part of the Identifying Optimal Models of HIV Care and Treatment study. All data were de-identified before analysis and the investigators had no access to identifiable patient information. Institutional review board approval was obtained from the ethical review board in Kenya; the study was designated non-human subjects research by the institutional review board at Columbia University Medical Center and the Center for Global Health at the United States Centers for Disease Control and Prevention.

## Results

Table 1 presents the facility-level and patient-level characteristics of the population. The majority of facilities in this study were public primary health-care centres (33 facilities, 52%), treating 5856 (44%) patients. Nearly all facilities were situated in urban or semi-urban settings. CD4+ cell testing was performed on-site in 18 facilities (29%) where 5661 patients were enrolled (42% of the patient population). For the remaining facilities and patients, blood drawn at the facility was transported off-site for CD4+ cell testing. Community ART adherence groups were available at 32 facilities (51%), financial incentives for ART adherence at 5 facilities (8%) and group counselling at 61 facilities (97%). At 5 facilities (8%) of physicians were available in addition to nurses and other health-care workers, providing care to 1436 patients (11%); other facilities were staffed only by nurses and clinical officers. Outreach workers were available at 29 facilities (46%). Most patients were enrolled at facilities where nurses assessed patients for ART eligibility (11 996, 90%), initiated ART prescriptions (11 092, 83%) and gave follow-up care to patients on ART (10 738, 81%).

**Table 1:** Characteristics of facilities and patients in the study of quality of HIV care at health facilities in the Eastern and Nyanza regions of Kenya, January 2014 to March 2016

Characteristic	**No. (%) of facilities** **(*n* = 63)**	**No. (%) of patients enrolled** **(*n* = 13 331)**
**Facility characteristics**		
Facility type		
Public primary	33 (52)	5 856 (44)
Public secondary	24 (38)	6 662 (50)
Private^a^	6 (10)	813 (6)
Facility location		
Urban	1 (2)	650 (5)
Semi-urban	27 (43)	6 824 (51)
Rural	35 (56)	5 857 (44)
Region		
Eastern	16 (25)	3 636 (27)
Nyanza	47 (75)	9 695 (73)
CD4+ cell testing		
Specimen collected & analysed at facility	18 (29)	5 661 (42)
Specimen collected at facility, analysed elsewhere	45 (71)	7 670 (58)
Community ART adherence group		
Yes	32 (51)	6 871 (52)
No	31 (49)	6 460 (48)
Financial incentives for adherence		
Yes	5 (8)	1 346 (10)
No	58 (92)	11 985 (90)
Facility-based adherence support, group counselling		
Yes	61 (97)	13 038 (98)
No	2 (3)	293 (2)
Assessment of ART eligibility by nurse^b^		
Yes	56 (89)	11 996 (90)
No	7 (11)	1 335 (10)
Initiation of ART by nurse^b^		
Yes	54 (86)	11 092 (83)
No	9 (14)	2 239 (17)
Follow-up of ART by nurse^b^		
Yes	53 (84)	10 738 (81)
No	10 (16)	2 593 (19)
Physician at HIV clinic^c^		
Yes	5 (8)	1 436 (11)
No	58 (92)	11 895 (89)
Outreach workers at HIV clinic		
Yes	29 (46)	6 496 (49)
No	34 (54)	6 835 (51)
Patient volume, no. of new enrollees per year		
< 150	24 (38)	2 358 (18)
150–300	29 (46)	6 283 (47)
> 300	10 (16)	4 690 (35)
**Patient characteristics at enrolment**		
Median (IQR) age, years	N/A	30.2 (23.7–38.5)
Age, years		
< 2	N/A	415 (3)
2–14	N/A	836 (6)
15–24	N/A	2 652 (20)
25–39	N/A	6 530 (49)
40–49	N/A	1 727 (13)
50+	N/A	1 171 (9)
Sex		
Male	N/A	4 728 (35)
Female	N/A	8 603 (65)
Immune status at enrolment		
CD4+ cell count > 200 cells/µL, or WHO stage 1 or 2	N/A	7 847 (59)
CD4+ cell count ≤ 200 cells/µL, or WHO stage 3 or 4	N/A	3 612 (27)
Unrecorded	N/A	1 872 (14)

ART: antiretroviral therapy; CD4+ cell: CD4+ T lymphocyte; HIV: human immunodeficiency virus; IQR: interquartile range; N/A: not applicable; WHO: World Health Organization.

^a^ Private-sector facilities are not formally categorized into primary or secondary by the government of Kenya. Health centres listed as private were mostly faith-based health centres offering limited services similar to primary-care centres.

^b^ Nurse-led care included care by clinical officers (non-physician clinicians).

^c^ Facilities with physician-led care also provided care by nurses or clinical officers.

Notes: *n* is the total number of facilities or total number of patients enrolled at those facilities. Data were collected for patients enrolling in HIV care between January 2014 and March 2015 and followed up to 31 March 2016.

Of the patient-level characteristics investigated, median age at enrolment was 30.2 years (interquartile range, IQR: 23.7–38.5), and 8603 (65%) patients were female. At enrolment in HIV care, 7847 patients (59%) had a CD4+ cell count > 200 cells/µL, or WHO stage 1 or 2; 3612 (27%) had a CD4+ cell count < 200 cells/µL, or WHO stage 3 or 4; and 1872 (14%) were missing both CD4+ cell count and WHO stage. Of those with unrecorded immune status at enrolment, 36 (2%) were recorded as dead at 12 months, compared with 80 (1%) among those with a CD4+ cell count > 200 cells/µL, or WHO stage 1 or 2, and 136 (4%) among those with a CD4+ cell count ≤ 200 cells/µL, or WHO stage 3 or 4.

Table 2 shows the results of univariate analyses, presenting the relative risk of retention in comprehensive HIV care for 12 months after enrolment. Of the 13 331 patients in the study sample, 8404 (63%) achieved comprehensive retention in care. Of the 4927 (37%) not achieving comprehensive retention, 208 (4%) died within 12 months, 629 (13%) never had their ART eligibility assessed, 381 (8%) were ART-eligible but did not initiate therapy and 3709 (75%) were lost to follow-up. 

**Table 2:** Univariate analyses of comprehensive retention in care among 13 331 HIV-positive patients enrolled in care at 63 facilities in the Eastern and Nyanza regions of Kenya, January 2014 to March 2016

Variable	Total no. of patients	Patients retained in comprehensive care for 12 months,^a^ no. (%)		RR (95% CI)^b^
		Yes	No	
**Total**	13 331	8 404 (63)	4 927 (37)	N/A
**Facility type**				
Public primary	5 856	3 780 (65)	2 076 (35)	1.09 (0.93–1.28)
Public secondary	6 662	4 077 (61)	2 585 (39)	Ref.
Private	813	547 (67)	266 (33)	1.04 (0.79–1.37)
**Facility location**				
Urban	650	424 (65)	226 (35)	1.08 (0.60–1.97)
Semi-urban	6 824	4 456 (65)	2 368 (35)	1.08 (0.93–1.26)
Rural	5 857	3 524 (60)	2 333 (40)	Ref.
**Region**				
Eastern	3 636	2 491 (69)	1 145 (31)	1.12 (1.09–1.15)
Nyanza	9 695	5 913 (61)	3 782 (39)	Ref.
**CD4+ cell testing**				
Specimen collected & analysed at facility	5 661	3 581 (63)	2 080 (37)	0.98 (0.83–1.16)
Specimen collected at facility, analysed elsewhere	7 670	4 823 (63)	2 847 (37)	Ref.
**Community ART adherence group**				
Yes	6 871	4 432 (65)	2 439 (35)	1.02 (0.88–1.18)
No	6 460	3 972 (61)	2 488 (39)	Ref.
**Financial incentives for adherence**				
Yes	1 346	901 (67)	445 (33)	1.09 (0.83–1.44)
No	11 985	7 503 (63)	4 482 (37)	Ref.
**Adherence support: group counselling**				
Yes	13 038	8 218 (63)	4 820 (37)	1.15 (0.74–1.78)
No	293	186 (63)	107 (37)	Ref.
**Assessment of ART eligibility by nurse**				
Yes	11 996	7 829 (65)	4 167 (35)	1.27 (1.00–1.61)
No	1 335	575 (43)	760 (57)	Ref.
**Initiation of ART by nurse**				
Yes	11 092	7 318 (66)	3 774 (34)	1.30 (1.06–1.60)
No	2 239	1 086 (49)	1 153 (51)	Ref.
**Follow-up of ART by nurse **				
Yes	10 738	7 116 (66)	3 622 (34)	1.29 (1.06–1.58)
No	2 593	1 288 (50)	1 305 (50)	Ref.
**Physician at HIV clinic**				
Yes	1 436	649 (45)	787 (55)	0.75 (0.57–0.98)
No	11 895	7 755 (65)	4 140 (35)	Ref.
**Outreach workers at HIV clinic**				
Yes	6 496	4 122 (63)	2 374 (37)	0.96 (0.83–1.12)
No	6 835	4 282 (63)	2 553 (37)	Ref.
**Patient volume, no. of new enrollees per year**				
< 150	2 358	1 156 (45)	1 420 (55)	0.78 (0.65–0.93)
150–300	6 283	2 489 (64)	1 383 (36)	0.95 (0.80–1.12)
> 300	4 690	4 759 (69)	2 124 (31)	Ref.
**Age, years**				
< 2	415	251 (60)	164 (40)	0.95 (0.88–1.03)
2–14	836	559 (67)	277 (33)	1.08 (1.02–1.14)
15–24	2 652	1 689 (64)	963 (36)	1.01 (0.97–1.05)
25–39	6 530	4 108 (63)	2 422 (37)	Ref.
40–49	1 727	1 102 (64)	625 (36)	0.94 (0.89–0.99)
50+	1 171	695 (59)	476 (41)	0.99 (0.96–1.03)
**Sex**				
Male	4 728	2 940 (62)	1 788 (38)	0.97 (0.95–1.00)
Female	8 603	5 464 (64)	3 139 (36)	Ref.
**Immune status at enrolment**				
CD4+ cell count > 200 cells/µL, or WHO stage 1 or 2	7 847	5 674 (72)	2 173 (28)	Ref.
CD4+ cell count ≤ 200 cells/µL, or WHO stage 3 or 4	3 612	1927 (53)	1 685 (47)	0.75 (0.73–0.78)
Unrecorded	1 872	803 (43)	1 069 (57)	0.67 (0.64–0.71)

ART: antiretroviral therapy; CD4+ cell: CD4+ T lymphocyte; CI: confidence interval; HIV: human immunodeficiency virus; N/A: not applicable; Ref.: reference category; RR: relative risk; WHO: World Health Organization.

^a^ Patients achieved 12 months of retention in comprehensive HIV care if they were: assessed for ART eligibility (CD4+ cell count or WHO stage); initiated on ART, if eligible; and retained on ART for 12 months after enrolment, or retained in care for 12 months if ART ineligible.

^b^ Relative risks of achieving 12-month retention in care were calculated using generalized linear mixed log-binomial relative risk regression with random intercepts to account for within-clinic correlation.

Note: Data were collected for patients enrolling in HIV care between 1 January 2014 and 31 March 2015 and followed up to 31 March 2016.

Retention in comprehensive HIV care for 12 months was significantly more likely at facilities where nurses assessed patients for eligibility and initiated and followed-up patients on ART. For example, 66% (7318/11 092) of patients enrolled at facilities where nurses initiated ART were retained in comprehensive care for 12 months, compared with 49% (1086/2239) of patients at facilities where nurses did not initiate therapy (RR: 1.30; 95% CI: 1.06–1.60). In contrast, patients at facilities with a physician were less likely to achieve 12-month retention than those at facilities without a physician (RR: 0.75; 95% CI: 0.57–0.98). In addition, patients at facilities with the smallest new patient enrolment had significantly lower 12-month retention in comprehensive care (RR: 0.78; 95% CI: 0.65–0.93) compared with sites with the medium level of enrolment. Lastly, 12-month retention was significantly lower among patients with more advanced HIV disease (RR: 0.75; 95% CI: 0.73–0.78) and those missing CD4+ cell count or WHO staging (RR: 0.67; 95% CI: 0.64–0.71).

Table 3 shows the findings based on multivariate analyses. In model 1 (adjusting only for facility-level characteristics), patients at facilities where nurses initiated ART were significantly more likely to be retained in care for 12 months (RR: 1.25; 95% CI: 1.01–1.54). No other facility-level characteristics remained statistically significant in this model. In model 2, in which we adjusted for both facility- and patient-level characteristics, 12-month retention was more likely for patients attending facilities with nurse-initiated ART (RR: 1.22; 95% CI : 1.00–1.48) and less likely among patients having advanced HIV disease at enrolment (RR: 0.65; 95% CI: 0.60–0.70).

**Table 3:** Multivariate analysis of comprehensive retention in care among 13 331 HIV-positive patients enrolled in care at 63 facilities in the Eastern and Nyanza regions of Kenya, January 2014 to March 2016

Variable	Patients retained in comprehensive care for 12 months**^a^** (yes/no)	
	Model 1**^b^** ****RR**^c^** (95% CI)	Model 2**^b^** ****RR**^c^** (95% CI)
**Initiation of ART by nurse**^d^		
Yes	1.25 (1.01–1.54)	1.22 (1.00–1.48)
No	Ref.	Ref.
**Physician at HIV clinic**		
Yes	0.82 (0.61–1.10)	0.83 (0.64–1.09)
No	Ref.	Ref.
**Patient volume, no. of new enrollees per year**		
< 150	1.04 (0.84–1.30)	1.05 (0.86–1.28)
150–300	0.90 (0.73–1.12)	0.92 (0.76–1.12)
> 300	Ref.	Ref.
**Facility type**		
Public primary	1.04 (0.88–1.22)	0.98 (0.84–1.14)
Public secondary	Ref.	Ref.
Private	1.01 (0.77–1.32)	1.00 (0.96–1.05)
**Age, years**		
< 2	N/A	0.93 (0.81–1.06)
2–14	N/A	1.03 (0.93–1.15)
15–24	N/A	0.96 (0.89–1.04)
25–39	N/A	Ref.
40–49	N/A	0.93 (0.84–1.02)
50+	N/A	0.98 (0.91–1.04)
**Sex**		
Male	N/A	1.00 (0.96–1.05)
Female	N/A	Ref.
**Immune status at enrolment**		
CD4+ cell count > 200 cells/µL, or WHO stage 1 or 2	N/A	Ref.
CD4+ cell count ≤ 200 cells/µL, or WHO stage 3 or 4	N/A	0.65 (0.60–0.70)
Unrecorded	N/A	0.74 (0.70–0.78)

ART: antiretroviral therapy; CD4+ cell: CD4+ T lymphocyte; CI: confidence interval; HIV: human immunodeficiency virus; N/A: not applicable; Ref.: reference category; RR: relative risk; WHO: World Health Organization.

^a^ Patients achieved 12 months of retention in comprehensive HIV care if they were: assessed for ART eligibility (CD4+ cell count or WHO stage); initiated on ART, if eligible; and retained on ART for 12 months after enrolment, or retained in care for 12 months if ART ineligible.

^b^ Model 1 included only facility-level covariates. Model 2 included facility-level and patient-level covariates. Facility type was a priori identified as a potential confounder of the relationship between availability of ART initiation and management by a nurse and retention at 12 months. Other facility-level covariates found significant at α < 0.05 in the univariate model were included in the multivariable models. All three patient-level covariates were included in the models.

^c^ Relative risks of achieving 12-month retention in care were calculated using generalized linear mixed log-binomial relative risk regression with random intercepts to account for within-clinic correlation.

^d^ Because eligibility assessment by nurse, initiation of ART by nurse and follow-up of ART by nurse were highly collinear with each other, only nurse initiation of ART by nurse was included in the models.

Note: Data were collected for patients enrolling in HIV care between 1 January 2014 and 31 March 2015 and followed up to 31 March 2016.

## Discussion

In our analysis of 13 331 HIV-infected patients enrolled in HIV care in Kenya, we found that the majority of clinical services were provided by nurses and clinical officers. Nurse-led ART services were associated with significantly higher comprehensive retention in HIV care and treatment and this was the only site-level characteristic significantly associated with retention in the multivariate analysis. In addition, when adjusting for both facility- and patient-level characteristics, nurse-led ART services remained significantly associated with higher rates of comprehensive retention of patients in HIV care.

Previously published studies have shown that nurse-managed ART does not compromise, and may improve, the quality of care for people living with HIV. In a systematic review of task-shifting, eight studies showed that nurse-managed HIV services in low-resource settings had outcomes similar to physician-led teams.^13^ Two randomized studies from South Africa also support the effectiveness of nurse-managed ART. A study at two health facilities near Cape Town and Johannesburg demonstrated that patients randomized to ART initiated by a physician and then monitored by a primary-care nurse had outcomes that were not inferior to patients randomized to physician-managed treatment.^14^ In the other study, a cluster-randomized trial showed equivalence of nurse-initiated versus physician-initiated ART at 31 health facilities in the Free State province of South Africa.^15^ In comparison to the previously published literature, our study is distinguished by its use of data from a larger number of health facilities, inclusion of both patient- and facility-level characteristics, the use of a novel outcome measure of comprehensive retention and the use of routine programmatic data.

Our findings also highlight the scarcity of physicians at HIV care settings in Kenya, even in urban and peri-urban areas. Only 8% of facilities had physician-led care, and only 11% of patients were being managed by a medical doctor. This is consistent with the situation in most low-resource settings where nurses, clinical officers and other non-physician clinicians provide the majority of health-care services.^16^^–^^18^ In sub-Saharan Africa, where the burden of HIV is the highest, nurses are the backbone of the health system.^19^^,^^20^

The strengths of this study include its novel measurement of quality of care, which combines a measure of retention in care with indicators of appropriate patient monitoring and initiation of treatment. Other strengths include the large number of patients and health facilities, and the inclusion of both patient- and facility-level parameters. The limitations include the non-random sample of health facilities, which were also limited to one country. While we adjusted for multiple patient and facility-level characteristics, the findings could have been affected by other parameters that we did not measure in this non-randomized study. The study focused on retention of patients and provision of key services; other important quality parameters that were not routinely collected at the time, such as achievement of viral suppression, patient satisfaction and quality of life, were not assessed and require further research.

In conclusion, using a comprehensive multi-component measure of quality of care, we demonstrated that patients at facilities offering nurse-led ART services were more likely to be retained in effective HIV care and treatment at 12 months after adjusting for facility- and patient-level characteristics. These findings confirm the central role of nurses in the achievement of global targets, including the 90–90–90 goals, and the need for further investment in nursing education, training and mentoring.
